# Need for Expansion of Pharmacy Education Globally for the Growing Field of Nanomedicine

**DOI:** 10.3390/pharmacy10010017

**Published:** 2022-01-21

**Authors:** Amy E. Barton, Gerrit Borchard, Matthias G. Wacker, Giorgia Pastorin, Imran Y. Saleem, Shaqil Chaudary, Tamer Elbayoumi, Zhigang Zhao, Beat Flühmann

**Affiliations:** 1 Vifor Pharma Group, Vifor Pharma Management Ltd., Flughofstrasse 61, 8152 Glattbrugg, Switzerland; beat.fluehmann@viforpharma.com; 2 Section of Pharmaceutical Sciences, School of Pharmaceutical Sciences of Western Switzerland (ISPSO), University of Geneva,1, Rue Michel Servet, 1211 Geneva, Switzerland; Gerrit.Borchard@unige.ch; 3 Department of Pharmacy, Faculty of Science, National University of Singapore, 5 Science Drive 2, Singapore 117545, Singapore; matthias.g.wacker@nus.edu.sg (M.G.W.); phapg@nus.edu.sg (G.P.); 4 School of Pharmacy and Biomolecular Sciences, Liverpool John Moores University, Liverpool L3 3AF, UK; i.saleem@ljmu.ac.uk (I.Y.S.); s.chaudary@ljmu.ac.uk (S.C.); 5 Department of Pharmaceutical Sciences & Nanomedicine Center of Excellence, College of Pharmacy Glendale Campus, Midwestern University, 19555 N. 59th Avenue, Glendale, AZ 85308, USA; telbay@midwestern.edu; 6 Department of Clinical Pharmacy, School of Pharmacy, Capital Medical University, No.10, Xitoutiao, You’anmen Wai, Fengtai District, Beijing 100069, China; zhaozhigang@bjtth.org; 7 Department of Pharmacy, Beijing Tiantan Hospital, Capital Medical University, 119 Nansihuan Xi Lu, Fengtai District, Beijing 100070, China

**Keywords:** nanomedicine, pharmacy, pharmacist, liposomes, nanoparticles

## Abstract

The emerging landscape of nanomedicine includes a wide variety of active pharmaceutical ingredients and drug formulations. Their design provides nanomedicines with unique features leading to improved pharmacokinetics and pharmacodynamics. They are manufactured using conventional or biotechnological manufacturing processes. Their physical characteristics are vastly different from traditional small-molecule drugs. Pharmacists are important members of the multi-disciplinary team of scientists involved in their development and clinical application. Consequently, their training should lead to an understanding of the complexities associated with the production and evaluation of nanomedicines. Therefore, student pharmacists, post-doctoral researchers, and trainees should be given more exposure to this rapidly evolving class of therapeutics. This commentary will provide an overview of nanomedicine education within the selection of pharmacy programs globally, discuss the current regulatory challenges, and describe different approaches to incorporate nanomedicine science in pharmacy programs around the world.

## 1. Introduction

The rapid expansion of nanotechnology and nanomaterial science in the late 1990s established the nanomedicine domain as an independent scientific discipline [1]. Nearly half of the currently approved nanomedicines in the United States, for example, were approved in the last decade, which illustrates the rapid growth of these unique drug therapies [2]. Compared with their small-molecule parent drugs, nanomedicines are intentionally designed to circumvent biologic barriers and display targeted pharmacokinetic and pharmacodynamic profiles to enhance safety and therapeutic efficacy [1,3]. However, many challenges and research gaps remain to be addressed in the field of nanomedicines, including how best to characterize these agents, evaluate their complex pharmacokinetic and biodistribution profiles, and compare their cost-benefit ratios to those of small-molecule drugs [3,4]. Pharmacists are the healthcare professionals with the most critical role in both clinical and administrative decisions related to the use of nanomedicines. They are also the best-trained healthcare professionals with regard to the fundamental concepts underlying the complex class of nanomedicines. With the ongoing evolution of nanomedicines of direct relevance to pharmacists working in distribution, clinical practice, research, and pharmacy administration, an innovative, forward-looking approach should be employed to incorporate nanomedicine content into pharmacy curricula.

## 2. The Nanomedicine Science Domain

**Figure 1:**
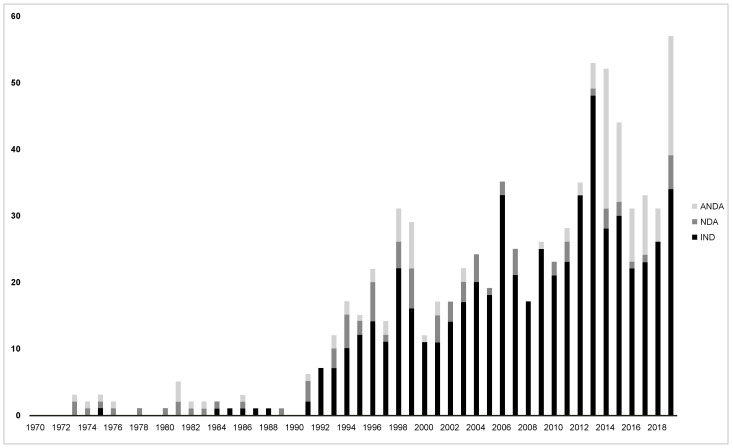
Human drug product submissions to the FDA containing nanomaterials, 1970–2019, Adapted from [2]. ANDA, abbreviated new drug application; NDA, new drug application; IND, investigational new drug application.

Although the term “nanomedicine” was likely coined in the early 1970s, interest in the nanomedicine science domain has only more recently shown an accelerated trajectory, exemplified by the rapid rise in regulatory submissions related to nanomedicines starting in the late 1990s (Figure 1).

In 2000, the United States National Institutes of Health launched the National Nanotechnology Initiative as a federal program to accelerate research and development [2]. Most regulatory agencies define a nanomedicine or a drug product containing nanomaterials as a preparation or formulation containing individual nanoparticles with dimensions of between approximately 1 and 100 nanometers (nm), where different physicochemical qualities and biological interactions confer unique qualities or phenomena attributable to these dimensions [5,6]. In addition to the nanomedicines currently in clinical use, the pipeline for new nanomedicines remains robust. According to an analysis by the Center for Drug Evaluation and Research (CDER, USA), there have been more than 600 investigational new drug (IND), new drug (NDA), and abbreviated new drug (ANDA) applications for human drug products containing nanomaterials [2]. The term “nanomedicines” broadly encompasses complex formulations that include nanoparticles in the form of liposomes, polymers, micelles, nanocrystals, inorganic metal-carbohydrates, and protein–ligand-based drugs [1,3,4,7]. In the European Union (EU), many nanomaterial-related drug products have entered the market [8]. However, recent public controversy regarding the general risks and hazards associated with nanotechnology has overshadowed this progress [9,10]. For example, nanomaterial regulations have become part of the directive on Registration, Evaluation, Authorisation, and Restriction of Chemicals (REACH), with a considerable impact on the availability of excipients with nanomaterial-related properties in the EU market. One possible explanation for the growing general resistance to the application of nanomaterials is a lack of education. While nanomaterial emissions pose a certain risk to the environment, nanomedicine is often confronted by the same biases—even though this technology has been explored for many years and has a solid track record in terms of clinical safety [9,10].

**Figure 2:**
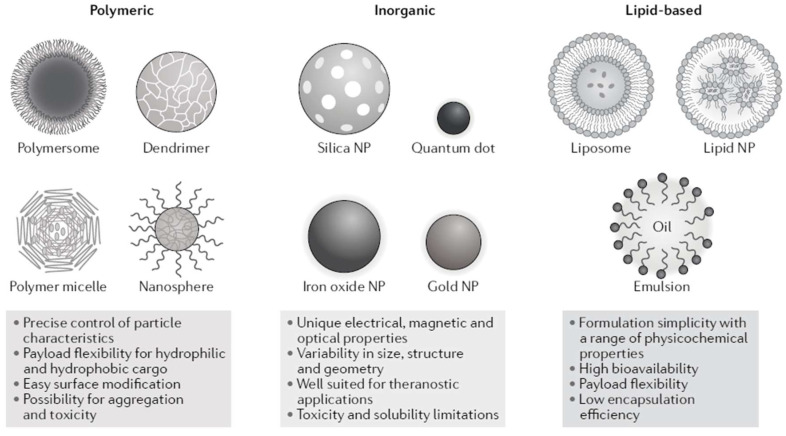
Common classes of nanomedicines [1].

A schematic overview of the major types of nanomedicines is presented in Figure 2. For a comprehensive discussion of their composition, readers are referred to several extensive reviews [1,4,7].

Over 50 nanomedicines have been approved for clinical use and are currently available on the global market [3]. The majority of nanomedicines approved for clinical use are based on liposome technology, most of which were developed to enhance tissue targeting in cancer therapy [3,11]. Liposomal encapsulated doxorubicin (“Doxil”) was approved by the United States Food and Drug Administration (US-FDA) in 1995 and is generally considered to be the first approved nanomedicine [12]. The liposomal doxorubicin formulation was designed to increase delivery to the tissue compartment and reduce exposure to the free drug, thereby reducing the cardiotoxicity of doxorubicin [3,12]. Other liposomal nanomedicine formulations include liposomal amphotericin B, cytarabine, and irinotecan. The first two approved vaccines for COVID-19 use messenger ribonucleic acid (mRNA) technology; their improved bioavailability and delivery to the cytosol is accomplished by complexing mRNA with positively charged lipids, forming lipid nanoparticles [13]. Polymeric nanomedicines contain proteins and polypeptides covalently linked to a varying number of polyethylene glycol (PEG) chains, and display greater stability and longer circulation times compared with their non-pegylated parent drugs. Polymeric nanomedicines include medications such as glatiramer acetate, as well as those containing a highly complex mixture of polypeptide chains [3]. Water-insoluble nanocrystals are generally designed to circumvent bioavailability issues related to the solubility of the crystal structure of the active pharmaceutical ingredient. The immunosuppressant sirolimus (Rapamune) is an example of a nanocrystal designed to produce better oral bioavailability compared with the oral solution [14]. Another group of nanomedicines is based on inorganic metal-carbohydrate nanoparticles, which protect the highly reactive metal ion from rapid dissolution in plasma [15]. The first iron-carbohydrate nanomedicine to treat iron deficiency—iron sucrose—was approved in Switzerland in 1949; however, it was not formally recognized as a nanomedicine at that time. Protein–drug conjugates utilize common proteins such as albumin as nanodrug carriers to improve solubility and targeted delivery to tissues (e.g., tumors) [1].

## 3. Challenges in Regulatory Evaluation of Nanomedicines

From a regulatory perspective, the evaluation of nanomedicines poses unique challenges compared with small-molecule drugs [16,17,18,19]. Only a limited number of validated physicochemical characterization methods exist at present due to the inherent properties, heterogeneity, and stability of nanomedicines. For example, the disassembly of nanomaterial-related drug products depends on a variety of experimental conditions such as the particle concentration and the availability of the free drug, ions, or proteins in the direct microenvironment of the carrier. This alters the structure in vitro and in vivo, and thus a need exists to establish reference standards for each drug/drug class [20,21,22]. Additionally, characterizing the in vivo behavior of nanomedicines is exceedingly challenging. As nanomedicines are designed to target specific tissues and cell types, plasma pharmacokinetics traditionally used in drug evaluation may not accurately describe tissue biodistribution, especially over a long time [23,24]. Moreover, conventional quantification methods do not accurately measure the circulating fraction of the drug bound to blood cells. Few assays can distinguish the free drug from the encapsulated drug, and target tissue deposition, biodegradation, and subsequent bioavailability may vary over several weeks [23,24]. The US-FDA identified eleven factors that constitute unique considerations and are fundamentally critical to the risk-based evaluation of drug products containing nanomaterials (Table 1) [6].

While these factors add challenge to the development and approval of new nanomedicines, the lack of data surrounding many of these identified factors naturally extends to the evaluation and approval process for follow-on copies of nanomedicines [16,17,25]. As the proprietary process of manufacturing individual nanomedicines restricts knowledge regarding product composition, it is nearly impossible to manufacture an exact copy of a reference listed drug [20]. However, despite these challenges, many product-specific guidance documents from the US-FDA do not propose bioequivalence evaluation methods that cover all of the proposed factors. A study analyzing the regulatory approach of EU agencies revealed that, after 2015, a totality of evidence approach was used—not following the generic approval pathway 10(1), but rather the hybrid application 10(3) [26]. Therefore, pharmacists and pharmaceutical scientists in training must learn about the key gaps in regulatory science for nanomedicines and bring these skills into their clinical practice and research programs. Pharmacists also need to be aware of new scientific discoveries that affect the regulatory evaluation of nanomedicines in the drug development pipeline [1,27]. Pharmacists are the primary members of interdisciplinary teams with adequate training in pharmaceutical science, pharmacokinetics, and regulatory science to synthesize data on known research gaps and new discoveries and then apply these concepts to clinical therapeutic decision making, formulary management, and research investigations [28].

## 4. Nanomedicine Coverage in Pharmacy Education

Engineered nanomaterials are one of the most rapidly evolving domains in drug development and hold great potential to circumvent heterogeneous biological barriers and realize the fundamental goals of drug delivery for precision medicine [1]. Thus, regulatory approval and the aforementioned regulatory challenges associated with nanomedicine drug products are projected to grow exponentially [1,29]. There is an upward trajectory in the development of nanomedicines for clinical applications addressing complex disease states related to immunotherapy and genome engineering [1].

Current and future pharmacists need to be better familiarized and equipped with essential knowledge of nanotechnology to engage in the developing demands and challenges in their professional practices and research enterprises [27]. Fundamentally, the current educational reform within professional pharmacy programs is focused on the integration of fundamental science and clinical science education to ensure that students and trainees can apply concepts bidirectionally from bench to bedside (and back). There is a relative dearth of information regarding the rate and extent to which programs cover the medical application of nanotechnology in current pharmacy and pharmaceutical sciences curricula. An evaluation of the inclusion of nanotechnology in Master of Pharmaceutical Sciences programs in Portugal collected the curricula delivered at five public and four private Universities [30]. Curricula were systematically searched for references to nanotechnology or related terms (e.g., liposome, nano, colloid). References to nanotechnology were weighted differently based on a number of factors (e.g., whether the reference was in a curricular unit name versus in a single lecture). None of the universities surveyed offered courses with terms related to nanomedicine as an entire curricular unit. The number of nanotechnology topics or subtopics ranged from 1 to 11 across the nine programs. Colloids, micelles, and liposomes were among the top terms identified during this systematic review. The authors concluded that the coverage of nanomedicine-related topics was quite low overall and was highly heterogeneous among the Portuguese universities. These results are demonstrative of the need to evaluate the nanotechnology topics included in pharmacy and pharmaceutical sciences programs and provide a unique opportunity to further evaluate programs globally and propose a plan to harmonize key educational concepts.

## 5. Opportunities to Incorporate Nanomedicine Educational Content: A Global Snapshot

### 5.1. Studies in Pharmacy and Pharmaceutical Sciences in Switzerland

Academic training in pharmacy and pharmaceutical sciences in Switzerland is offered at cantonal universities (Basel, Berne, Geneva) and the Swiss Federal Institute of Technology (ETH) in Zürich. At all institutions, the program consists of a three-year Bachelor’s degree followed by a two-year Master’s phase, including a Master’s project. The pharmacy curricula offered at these institutions are formally based on the common “catalog of learning objectives” [31]. Students graduate with a Master of Science from their local institution and are accredited as licensed pharmacists after passing a federal exam. Several Master and Certificate of Advanced Studies (MAS/CAS) postgraduate programs are offered by different schools. In addition, the School of Pharmaceutical Sciences at the University of Geneva, in cooperation with the Faculty of Medicine, is organizing a BSc/MSc curriculum in Biomedical Sciences where graduates are trained to take over responsibilities in the pharmaceutical and biotechnology industries.

#### Nanomedicine Education at the University of Geneva’s School of Pharmacy

According to the recent QS ranking, the School of Pharmaceutical Sciences of Western Switzerland (ISPSO) at the University of Geneva is ranked number 33 of all schools of pharmacology and pharmacy worldwide, number 20 for its academic reputation, and number 1 of all French-speaking schools [32]. The pharmacy curriculum in Switzerland is determined by the federal catalog of learning objectives. However, within the general framework provided by the catalog, each school organizes the structure and content of its programs individually. The teaching philosophy at Geneva is based on the dichotomy of fundamental and clinical pharmaceutical sciences, and the conviction that excellence in teaching is determined by excellence in scientific research. Therefore, the respective research activities of the school’s different units influence the contents of the curriculum. The study program is submitted for accreditation by an independent organization, is under constant review and reform, and applies modern pedagogic tools such as inverted classrooms, objective structured clinical examination (OSCE), and simulations.

The catalog mentions “drug delivery systems” and “drug targeting” as topics to be taught to confer theoretical and practical competencies to students at the highest level. At ISPSO, several research groups focus on the development, characterization, and application of nanomedicine(s) for therapeutic and preventive purposes. Students are acquainted with nanomedicine(s), their development, characterization, application, pharmacokinetics, and related regulatory issues during the fourth year (the first year of the Master’s phase) within the module on “Drug Development”—especially in a lecture series on “complex drugs” given by the author. The new Master’s program, which was introduced in the fall of 2021, features courses dedicated to “colloidal vector systems”, “drug targeting”, and “advanced therapy medicinal products” (ATMP). Students can also choose to attend an optional course on nanomedicines offered in cooperation with the Faculty of Medicine. Hands-on training on the preparation of nanomedicines and their characterization, however, is not taught in lab courses to all students. The major reasons for this lack of practical training are the growing number of students and a lack of the dedicated, and expensive, equipment that is required. Practical student experience regarding nanomedicine is therefore currently restricted to Master’s projects in the units related to this domain.

### 5.2. Studies in Pharmacy and the Pharmaceutical Sciences in Singapore

The National University of Singapore (NUS) is the country’s flagship university with more than 30,000 students and offers a global approach to education and research, with a focus on Asian perspectives. In the QS world university rankings, it is ranked number 1 in Asia and number 11 worldwide [32]. NUS is the only institution offering training in Pharmacy and the Pharmaceutical Sciences in Singapore and offers two full-time four-year degree programs at the undergraduate level, namely the Bachelor of Pharmacy (Honours) program and the BSc (Pharmaceutical Science) (Honours) program.

#### Nanomedicine Education at NUS

During their final year, undergraduates have the opportunity to work on a nanomedicine project focusing on formulation techniques, release profiles, and proof-of-concept efficacy studies. These projects are offered twice a year and are immediately followed or preceded by pre-registration internships to become pharmacists in Singapore. Stronger integration of different domains began with the introduction of the new curriculum in 2020. Collaborative teaching strategies led to patient-centric education, which puts more emphasis on the Singaporean job market for pharmacists. In the new curriculum, one module is dedicated to “Creating the Future of Pharmacy”, which may be a suitable way in which to familiarize undergraduate students with nanomedicine-related topics; however, several other trends in academic research are presented under this umbrella.

Moreover, new design strategies for the development of drug formulations, including nanomedicines, are introduced as part of the “Advances in drug delivery” module for graduate students. The module is dedicated to a discussion of the recent progress in this research area. Students learn about the key concepts underlying nanomedicine, as well as the translational aspects that led to the application of nanomaterials in diagnosis and drug therapy. In the 21st century, we are now witnessing the development of the Nano-Age, with improvements to both the diagnosis of diseases and the pharmacological properties of conventional drugs. Despite such advances, the applications and advantages of nanomedicines continue to present challenges to healthcare professionals around the world.

Therefore, the course provides the knowledge required to understand nanotechnology at a deeper level and inspires graduate students to critically evaluate the advantages and disadvantages of novel drug delivery strategies. This will slowly lead to a broader acceptance of nanotechnology and the application of novel approaches against enigmatic pathological conditions for the benefit of patients.

The learning outcomes of this graduate module can be broadly classified as follows:

1. To acquire the knowledge and critically examine the innovative approaches taken by pharmaceutical industries and scientists to develop efficient drug delivery systems and/or diagnostic devices.
2. To present a selected topic and share own knowledge on nanomedicine through small group discussions.
3. To be able to summarize key information and data from the literature in a concise, relevant, and conducive manner.

Toward these goals, the module is conducted through lectures (once a week) which include both conventional (lecture notes previously uploaded on the NUS server) and innovative (e.g., fish-bowl strategy) teaching modes. The “fishbowl” is a teaching strategy that helps students to be engaged in discussions in class, both as contributors and listeners. Students are assigned selected topics regarding the use of nanomedicines for drug delivery. According to the lecture slots, they ask questions, present opinions, and share information when they sit in the “fishbowl” (inner semi-circles), while students on the outside of the semi-circles listen carefully to the ideas presented. The roles reverse based on each student’s involvement in the discussion (i.e., students that participate actively are challenged with further questions, while the lecturer tries to engage with those students that did not have the chance to speak in front of the class). The decision to adopt this strategy for this module is due to two aspects:

(1) Availability of a small class (usually less than 50 students), which enables the easier organization of students and teaching material; and
(2) The intention is to have all students participate in discussions: in fact, even students that are sitting outside the semi-circles might be asked for their opinion at any point in time. This is perceived as particularly beneficial when we want to discuss controversial or difficult topics.

The purpose is to make students become “experts” on a topic and to feel they “own” it. This is possible by converting the role of the lecturer from the one “delivering” the lecture, to the one coordinating the discussion.

### 5.3. Studies in Pharmacy and the Pharmaceutical Sciences in the United Kingdom

Pharmacy education in Liverpool began in 1849 and is well established [33]. Liverpool John Moores University is amongst the oldest pharmacy education providers in the UK. The current four-year MPharm program was designed as a spiraling integrated program with input from all key stakeholders [34]. As such, scientific concepts and skills are developed year on year while increasing in complexity. The core of the MPharm program delivery includes lectures, laboratory practicals, and workshops, which cover strategies for formulating nanomedicines using examples of liposomal formulations currently on the market.

Student learning also involves current research perspectives on developments and challenges in nanomedicines. For example, students are taught about how nanomedicines can be used to aid in crossing the blood-brain barrier to treat Alzheimer’s disease, in nanocarrier systems for the delivery of biological payloads for cancer therapy, and in vaccine candidates including the novel mRNA COVID-19 vaccines [35]. Students are also kept up to date on current developments related to nanotechnology-based systems including polymeric nanoparticles, solid lipid nanoparticles, and liposomes, ensuring an understanding of their major applications and routes of administration. A particular focus is placed on the non-parenteral administration of nanocarrier-based systems including intranasal delivery of nanoparticles for gene delivery to the brain, pulmonary delivery of nanocarrier systems incorporating antimicrobials for treatment of lung infections, and antigens for mucosal vaccination. Students are also allowed within their final year project to undertake lab-based work focused on the formulation and characterization of nanocarrier delivery systems.

Students also have the option to pursue an MSc, especially those with an interest in the pharmaceutical and cosmetic industries. Here, more in-depth topics associated with advanced nanocarrier systems are discussed with example topics related to vaccines, cancer therapy, eye delivery, and advanced cosmetic nanoformulations. Delivery of material includes lectures, laboratory practicals, and workshops. In addition, students undertake a mini-project involving the development and characterization of dry powder nanocarrier systems. Further topics discuss the toxicity of nanocarriers. Moreover, external speakers are invited from the pharmaceutical and cosmetic industries to discuss the regulatory aspects of nanocarrier delivery systems. Finally, students undertake a three-month project, some in collaboration with industry, providing hands-on experience in various techniques of manufacturing and characterizing nanocarrier systems targeting specific administration routes and diseases or cosmetic outcomes.

### 5.4. Studies in Pharmacy and the Pharmaceutical Sciences in the United States

Since the Doctor of Pharmacy degree became a prerequisite for being licensed to practice pharmacy in the United States, US Pharm D programs have been primarily expanding their experiential and clinical curricular components—at both the introductory and advanced level—focusing on effective and safe medication utilization. Hence, pharmacy programs have been primarily designed to prepare graduates to be practice-ready, with students spending most of their training time on direct patient care and clinical outcomes.

Recently, part of the current educational reform within professional pharmacy programs aims to effectively integrate foundational science and clinical science education. The 2016 standards from the Accreditation Council for Pharmacy Education (ACPE) emphasize pharmaceutical science knowledge as an essential framework for understanding clinical knowledge [36]. However, until now, the knowledge and fundamentals of nanoscale surface effects, quantum mechanical nanoscience, and the corresponding applied nanomaterials and nanotechnological applications are barely mentioned within the US Pharm D curriculum (only as a small part of introductory theoretical pharmaceutics courses). It merely exists in the form of independent dedicated theoretical courses and practical laboratory training and is only offered through dual degree programs, such as some of the Pharm D/PhD Pharmaceutical Science programs primarily available at R01 US universities, and in just a few of the many dual Pharm D/MS programs (Table S1) [37].

Focus on the unique technological opportunities ahead echoes through the ongoing American Association of Colleges of Pharmacy (AACP) strategic plan 2018–2021, where proposals for expanding research and graduate education were adopted as strategic priority number 4 [38]. For instance, nanopharmaceutical product development and evaluation research will not only support a more translational approach of pharmacy professionals toward patient care, but will also broaden career opportunities, especially given that the most significant sources of job growth outside of retail and hospital settings are managed care organizations, technology/biotech/pharmaceutical companies, and ambulatory care practices [37,39].

### Midwestern University, Glendale, Arizona

Data show that academic courses are the leading source of information, and pharmacy students, in general, show poor knowledge about fundamental nanotechnology science and its applications. Hence, there is an impending need to effectively incorporate education and training in nanomedicines and nanodiagnostics in curricular and co-curricular pharmacy courses and activities, respectively [40,41,42]. At Midwestern University (MWU), the College of Pharmacy (COP) only offers a year-round, three-year Pharm D program, where graduates receive the standard Doctor of Pharmacy degree (no other graduate/professional/certificate degrees are included or integrated). The establishment of the multidisciplinary MWU Nanomedicine Center of Excellence (COE) at COP in 2017 has allowed our teaching-focused college to become more competitive in its collaborative research and training programs. The student teaching and training components of the Nanomedicine COE, including the opportunity to create a nanoscale drug delivery research project and training elective courses, as well as a theoretical nanomedicine-structured elective course, are all integrated into the Pharm D curriculum at COP. Offered to pharmacy students in their second year, our entire “Nanopharmaceuticals” elective course focuses on nanoscale drug formulations and solid therapeutic nanoparticles, as well as nanoparticulate contrast and theranostic agents. Participants in this course become familiar with state-of-the-art pharmaceutical nanotechnology, based on comparisons between conventional drug therapies and FDA-approved nanopharmaceutical products. Building on related introductory nanoscience topics, the students learn about key therapeutic aspects (e.g., enhanced efficacy/pharmacodynamics, pharmacokinetics, and safety profiles) of several example nanomedicines currently used in clinical practice. This course aims to provide students with a foundation to realize the expected changes to therapy and diagnostics being brought about by pharmaceutical nanotechnology, thereby impacting their future careers.

The US healthcare system has developed dramatically over the past decade, where significant changes in the drug distribution system, automation/robotics, and advanced targeted/complex nanoscale therapeutic approaches were acknowledged as eminent technological disrupters, while the main drivers of US pharmacy education still involve a primary care provider shortage, rising medication/medical service costs, and the growing application of technology and data analysis [42]. To maintain the pharmacist’s essential role in the rapidly advancing medical marketplace, academic pharmacy education and post-graduate training need to adapt and adopt multi-faceted technological sciences and their applications, such as nanotechnology, biotechnology/precision medicine, bio/material science, and robotics/3D printing [43]. Such advanced Pharm D curricula will allow graduating pharmacists to grow as an integral part of the multidisciplinary healthcare team of tomorrow. Otherwise, future US pharmacy professionals risk being sidelined or even replaced by novel and smart healthcare systems, more capable of meeting patients’ clinical and pharmaceutical needs [27].

### 5.5. Studies in Pharmacy and the Pharmaceutical Sciences in China

At present, there are generally no colleges and universities in China that offer separate nanomedicine courses, but many have introduced nanomedicine concepts in relevant courses (e.g., pharmaceutics, advances in medicine). Courses related to peptides, small molecules, and liposomes are available in many colleges and universities. For example, the School of Pharmacy of Capital Medical University is home to the Beijing Key Laboratory of Peptide and Small-Molecule Drugs. In the studies for master and doctorate degrees, some pharmacy students will complete a graduation project with the help of elective courses. One such course, entitled Liposome Preparation, and Verification of Effect explores new methods of administration to improve the therapeutic effect or reduce adverse reactions to small-molecule drugs.

In 2002, the National Nano Initiative, proposed by the National Science Foundation, initiated a program to promote different levels of student education activities for nanoscience. Multidisciplinary nanoscience programs have also been implemented at major research universities in the United States, involving physical sciences, life sciences, and engineering. Various applications of nanoscience in the medical field have led to the continuous development of the subfield of nanomedicine in China. Over the past few years, academic journals, textbooks, and other professional literature for basic science research and pioneering clinical development in nanomedicine have increased, and a nanomedicine curriculum is also in the early stages of development [40,43].

Currently, the educational model of nanomedicine-related courses for undergraduate pharmacy students in China is still traditional and fundamentally focused on basic science. The experiments carried out only involve the preparation of simple liposomes or microspheres, and the characterization instruments used are also limited to optical microscopes. Nanomedicine is an interdisciplinary field, which has developed rapidly as an emerging technology, so diversification and cutting-edge techniques should be considered in the curriculum setting. Universities in China can also strengthen the joint construction of teaching and scientific research laboratories or innovative enterprises, allowing students to have access to large-scale precision instruments in the field of nanotechnology, apply theoretical knowledge in practice, and cultivate professional thinking, innovation, and hands-on ability, all of which facilitate the student’s ability to understand practical applications. In future evaluation systems, students’ mastery and application of “nanomedicine” can be examined through contemporary pedagogical approaches including process assessment, group discussion, participation in lectures, open papers, innovative experiments, and online learning. Table S2 summarizes the characteristics of some programs offering nanomedicine-focused curricula.

## 6. Summary on the Importance of Nanomedicine Education for Future Pharmacists

Strong foundations in the pharmaceutical sciences, together with the knowledge and skills required to understand the physiological, preclinical, and clinical aspects of drug development, have defined pharmacy education for generations. While most other disciplines require a much higher degree of specialization, the translation from bench to bedside was a centerpiece of pharmaceutical research long before it became a major trend in several scientific communities. With regard to nanomedicine, the gaps between those disciplines have become more apparent and mandate interdisciplinary research and approaches to education. Therefore, new integrated curricula present an opportunity to bridge critical gaps in the education of both future pharmacists and research scientists. However, in this context, the balance between a more clinically oriented education and fundamental scientific knowledge will be of utmost importance. Although we acknowledge the specialization that is offered at the graduate level, the scientific background of nanomedicine requires further coverage at the undergraduate level. Notably, the undergraduate and Doctor of Pharmacy curricula are already very content-heavy to sufficiently cover all required material for accreditation of the programs. Thus, innovative co-curricular electives and experiential courses can offer a highly effective means to deliver more advanced nanomedicine topics [27]. It is the authors’ collective sentiment that omissions of curricular content should be assessed and made by the relevant accrediting bodies, as these recommendations are country-specific. Nanotechnology knowledge integration with existing mandated pharmaceutical and clinical science topics can be explored at both curricular and co-curricular levels. Key concepts of nanomedicine effects on pharmacokinetics and pharmacodynamics can pragmatically emphasize the behavior of the material at the nanoscale. For example, almost 10% of oncology drugs available today are nanomedicines, and the enhanced tumor delivery of nanomedicines can be illustrated in relevant pharmacology and pharmacotherapy content via commercially approved nanomedicines, such as Doxil. A current trend in pharmacy education is the delivery of optional track programs with focused elective courses that build a framework for sub-specialty knowledge [44]. Elective courses and joint (e.g., industrial–clinical) practical experiences for interested students that are focused on the design and clinical applications of nanotechnology-based medical products could be offered to supplement nanomedicine concepts presented in the core curriculum [38,40,41,45]. A variety of other co-curricular activities could be developed by faculty to further integrate basic science nanomedicine concepts with clinical decision-making. These may include early non-patient-care-related interprofessional education/collaboration/innovation (e.g., research activities, independent study), medical technology-focused seminar series, or professional service organizations such as the Industry Pharmacist Organization (IPhO) student chapters [41,45].

The nanomedicine domain is expanding at a rapid rate. Because of the complexity of this class of drugs, pharmacists represent the discipline with the best aptitude to integrate pharmaceutical science, the regulatory evaluation, and clinical data to lead decision-making for patient care. Given the application of nanomedicines to a wide and diverse array of disease states, Doctor of Pharmacy curricula should be forward-thinking in their approach to including and expanding the coverage of nanomedicines. Given the density of material in pharmacy programs, innovative techniques such as co-curricular electives, student organization initiatives, and certificate programs can effectively provide didactic education on key nanomedicine concepts.

## Figures and Tables

**Table 1:** Factors crucial to the risk-based evaluation of drug products containing nanomaterials, adapted from [6,16].

Adequacy of Characterization of the Material Structure and Its Function
Complexity of the material structure
Understanding the mechanism by which the physicochemical properties of the material impact its biological effects (e.g., effect of particle size on pharmacokinetic parameters)
Understanding the in vivo release mechanism based on the material physicochemical properties
Predictability of in vivo release based on established in vitro release methods
Physical and chemical stability
Maturity of the nanotechnology (including manufacturing and analytical methods)
Potential impact of manufacturing changes, including in-process controls and the robustness of the control strategy on critical quality attributes * of the drug product
Physical state of the material upon administration
Route of administration
Dissolution, bioavailability, distribution, biodegradation, accumulation, and their predictability based on physicochemical parameters and animal studies

* Critical quality attributes have not been fully defined for all drug products containing nanomaterials.

## Data Availability

Not applicable.

## Supplementary Materials

The following supporting information can be downloaded at: https://www.mdpi.com/article/10.3390/pharmacy10010017/s1, Table S1: US pharmacy dual degree programs and nanomedicine focus (*programs that include nanomedicine topics) [45]; Table S2: Characteristics of a selection of global programs offering nanomedicine education.
