# Supplementary material for: Need for Expansion of Pharmacy Education Globally for the Growing Field of Nanomedicine
Source: Pharmacy (Basel). 2022 Jan 21;10(1):17. doi: 10.3390/pharmacy10010017 (PMC8878512; doi:10.3390/pharmacy10010017)
Supplement: Supplementary file 1 [file pharmacy-10-00017-s001.zip › pharmacy-1491833-supplementary.pdf]

Table S1. US pharmacy dual degree programs and nanomedicine focus (*programs that include nanomedicine topics)Adapted from [37].

Institution	Program	Dual Degree Program	
		MS	PhD
Albany College of Pharmacy and Health Sciences		1*	
Auburn University	Harrison School of Pharmacy		1*
Campbell U	College of Pharmacy & Health Sciences	1	
Creighton U	School of Pharmacy and Health Professions	1	
Drake U	College of Pharmacy & Health Sciences	1*	
Duquesne U	School of Pharmacy	1	
Fairleigh Dickinson U	School of Pharmacy	1	
Idaho St U - AK	College of Pharmacy		1*
Idaho St U	College of Pharmacy		1*
Lipscomb U	College of Pharmacy	1	
Loma Linda U	School of Pharmacy	1*	
Long Island U	Arnold & Marie Schwartz College of Pharmacy and Health Sciences	1*	
Manchester U	Pharmacy Program	1*	
Med U South Carolina	College of Pharmacy	1*	1*
Mercer U	College of Pharmacy	1*	1*
North Dakota St U	School of Pharmacy		1*
Oregon St U	College of Pharmacy		1*
Pacific U Oregon	School of Pharmacy	1*	
PCOM - GA	School of Pharmacy	1	
Regis U	Rueckert-Hartman College for Health Professions	1	
Rosalind Franklin U Med Sci	College of Pharmacy		1*
Samford U	McWhorter School of Pharmacy	1	
South Dakota St U	College of Pharmacy and Allied Health Professions		1*
Southern Illinois Edwardsville	School of Pharmacy	1	
Temple U	School of Pharmacy	1	
Ohio St U, The	College of Pharmacy		1*

U of Arizona, The	College of Pharmacy	1*	1*
U of Findlay, The	College of Pharmacy	1	
U of Iowa, The	College of Pharmacy	1	
U of Kansas, The	School of Pharmacy	1	
U of Louisiana Monroe, The	College of Pharmacy		1*
U of New Mexico, The	Health Sciences Center College of Pharmacy	1*	
U of Rhode Island, The	College of Pharmacy	1*	
U of Tennessee, The	College of Pharmacy	1*	1*
U of Texas Austin, The	College of Pharmacy		1^
U of Toledo, The	College of Pharmacy and Pharmaceutical Sciences	1	1*
U at Buffalo	School of Pharmacy and Pharmaceutical Sciences	1*	1*
U of Arkansas Med Sci	College of Pharmacy	1	1*
U of California, San Diego	Skaggs School of Pharmacy and Pharmaceutical Sciences		1*
U of California, San Francisco	School of Pharmacy		1*
U of Connecticut	School of Pharmacy		1*
U of Houston	College of Pharmacy	1*	1*
U of Illinois Chicago	College of Pharmacy	1*	1*
U of Kentucky	College of Pharmacy	1*	
U of Maryland	School of Pharmacy	1*	1*
U of Minnesota	College of Pharmacy	1	
U of Montana	Skaggs School of Pharmacy	1*	1
U of North Texas HSC	System College of Pharmacy	1*	1*
U of South Florida	School of Pharmacy	1	
U of Southern California	School of Pharmacy John Stauffer Pharmaceutical Sciences Center	1	1*
U of the Pacific	Thomas J. Long School of Pharmacy	1*	1
U of the Pacific - Pre-Pharm		1	1*
U of Washington	School of Pharmacy	1*	1*

Virginia Commonwealth U	School of Pharmacy	1*	1*
Washington St U	College of Pharmacy		1*
Wayne St U	College of Pharmacy		1*

Table S2. Characteristics of a selection of global programs offering nanomedicine education.

Program (Country)	Number of students	Degree programs offered*	Research opportunities offered	Student pharmacist careers post graduation
Unige (Switzerland)	100–150 per year	Bachelor of Pharmaceutical Sciences, Master in Pharmacy, Master in Biomedical Sciences	Master/PhD programmes	Public pharmacies, industry, hospitals, academic research institutes, international organisations (e.g., WHO, UN), regulatory bodies (Swissmedic, EMA)
NUS (Singapore)	Up to 50 students per year<br>About 150 students per year<br>About 150 students per year	Course in Advances in Drug Delivery (Pharmacy Department, NUS)<br>Bachelor of Engineering (Engineering Science) Specialisation in Nanoscience and Nanotechnology<br>(Bachelor of Engineering (Materials Science and Engineering) - Nanostructured Materials and Nanotechnology)	Part of the PhD and Master programmes<br>Final Year Projects for undergraduate students (mainly in Pharmacy, Chemistry and Engineering)	Health Science Authorities (HSA) Singapore (on regulatory aspects)<br>Research Institutes: Institute of Bioengineering and Nanotechnology (IBN)<br>Singapore Nanotech companies:<br>Advanced Materials Technologies Pte Ltd.<br>NanoGlobe<br>Malvern Panalytical (instrumentation) etc.<br>Nanotechnology Startups:<br>Salus Nano<br>Matralix<br>Peregrinephthalmic<br>Ophthalmic<br>PL Nanotechnolog<br>NanoMaterials Technology<br>NanoSun

Liverpool John Moores (UK)	150 per year	Masters in Pharmacy (MPharm)	Principles of research developed across all years culminating in a final year research project.	Undertake a 1-year Foundation training year (pre-registration year) and after successful completion become registered as pharmacists by the General Pharmaceutical Council (GPhC). In addition, students have an option to study MSc programs within the School.
	About 60–85 students	MSc Cosmetic Science (30-45 students)<br>MSc Drug Discovery, Development and Delivery (15-20 students)<br>MSc Natural Products Discovery (15-20 students)	3-month project with opportunities to undertake projects with & within companies	Career opportunities<br>Product Manufacturing<br>Regulatory & Legislation<br>Research & Development<br>Marketing & Technical
